# Chronic heart failure and mortality in patients with community-acquired *Staphylococcus aureus* bacteremia: a population-based cohort study

**DOI:** 10.1186/s12879-016-1570-7

**Published:** 2016-05-25

**Authors:** Jesper Smit, Kasper Adelborg, Reimar Wernich Thomsen, Mette Søgaard, Henrik Carl Schønheyder

**Affiliations:** Department of Clinical Microbiology, Aalborg University Hospital, Hobrovej 18-22, DK-9000 Aalborg, Denmark; Department of Infectious Diseases, Aalborg University Hospital, Mølleparkvej 4, P.O. Box 365, DK-9100 Aalborg, Denmark; Department of Clinical Epidemiology, Aarhus University Hospital, Olof Palmes Allé 43-45, DK-8200 Aarhus, Denmark; Department of Cardiology, Aarhus University Hospital, Palle Juul-Jensens Boulevard 99, DK-8200 Aarhus, Denmark; Department of Clinical Medicine, Aalborg University, Sdr. Skovvej 15, DK-9000 Aalborg, Denmark

**Keywords:** Congestive heart failure, *Staphylococcus aureus*, Bacteremia, Mortality, Prognosis

## Abstract

**Background:**

Patients with chronic heart failure (CHF) may experience higher mortality of *Staphylococcus aureus* bacteremia (SAB) than patients without CHF due to insufficient cardiovascular responses during systemic infection. We investigated 90-day mortality in SAB patients with and without CHF.

**Methods:**

Using population-based medical databases, we conducted a cohort study of all adult patients with community-acquired SAB (CA-SAB) in Northern Denmark, 2000-2011. Ninety-day mortality after SAB for patients with and without CHF was estimated by the Kaplan-Meier method. Based on Cox regression analysis, we computed hazard ratios as estimates of mortality rate ratios (MRRs) overall and stratified by CHF-related conditions (e.g., cardiomyopathy and valvular heart disease), CHF severity (defined by daily dosage of loop-diuretics), and CHF duration while adjusting for potential confounders.

**Results:**

Among 2638 SAB patients, 390 (14.8 %) had a history of CHF. Ninety-day mortality was 45 % in patients with CHF and 30 % in patients without CHF, which yielded an adjusted MRR (aMRR) of 1.24 (95 % CI, 1.04-1.48). Compared to patients without CHF, the excess risk of death was most pronounced among patients with valvular heart disease (aMRR = 1.73 (95 % CI, 1.26–2.38)), patients with daily loop-diuretic dosages of 81–159 mg/day (aMRR = 1.55 (95 % CI, 1.11–2.14)) and ≥160 mg/day (aMRR = 1.62 (95 % CI, 1.21–2.18)), and among patients with <3 years of CHF duration (aMRR = 1.43 (95 % CI, 1.14–1.78)).

**Conclusion:**

CA-SAB patients with CHF experienced increased 90-day mortality compared to patients without CHF.

**Electronic supplementary material:**

The online version of this article (doi:10.1186/s12879-016-1570-7) contains supplementary material, which is available to authorized users.

## Background

*Staphylococcus aureus* bacteremia (SAB) continues to be associated with considerable morbidity and a 30-day mortality of 20–40 % in developed countries [1, 2]. Chronic heart failure (CHF) currently affects more than 23 million persons worldwide, and hospitalizations and readmissions for CHF remain a major public health problem [3, 4]. Patients with CHF may experience higher mortality from SAB than patients without CHF due to insufficient cardiovascular responses to severe systemic infection [5]. Further, CHF and SAB share several negative prognostic factors including male sex, high age, and comorbidity [2].

Still, data on the prognostic impact of CHF in patients with SAB are limited and inconsistent, and to our knowledge no prior prognostic study has addressed CHF as the main exposure in this clinical setting. Previous studies have been restricted by a limited number of SAB patients (N < 400) [6–9], often from referral centers [9], and CHF has not been defined according to strictly specified criteria [6–9]. Other limitations include incomplete information on comorbid conditions [8, 9], and lack of follow-up after discharge [8, 9]. Detailed information on the prognostic influence of CHF in patients with SAB may extend our understanding of the clinical course of SAB patients and contribute to improved treatment for patients with CHF. Therefore, we conducted a population-based cohort study to examine the prognostic impact of CHF in patients with community-acquired SAB (CA-SAB).

## Methods

### Setting

This cohort study was conducted using routinely recorded data from population-based medical registries in Northern Denmark between 1 January 2000 and 31 December 2011 (catchment population ~ 1.8 million inhabitants). Tax-supported, unfettered healthcare is provided for the entire Danish population through a national health insurance program [10, 11]. Northern Denmark is served by two University hospitals and a dwindling number of regional hospitals (22 regional hospitals in 2000 and 7 regional hospitals in 2011). All Danish residents are assigned a unique identification number which allows unambiguous linkage of registry data at the individual level [10, 11].

### Patients with *S.aureus* bacteremia

Using the databases of the departments of clinical microbiology within the area, we identified all patients hospitalized with CA-SAB from 1995 onwards. We included patients ≥15 years with ≥1 positive blood cultures with *S.aureus* as the sole isolate (information on blood culture practice and susceptibility testing is provided in Additional file 1: Identification and susceptibility testing of *S. aureus* isolates). Because recurrence of SAB may affect prognosis [12], we limited the study to patients with incident SAB, defined as no prior SAB diagnosis within at least 5 years of the current hospitalization.

CA-SAB was defined as SAB in patients, in whom one or more positive blood cultures had been obtained within the first two days of admission. Patients with a first blood cultured obtained >2 days after admission were excluded, because we consider these infections to be hospital-acquired. Patients with CA-SAB and healthcare contacts recently preceding the current admission were sub-classified as healthcare-associated SAB (HCA-SAB) if one or more of the following criteria were met: ≥1 hospital admission, ≥1 contacts to hospital outpatient surgical clinics (including minor surgery), or ≥1 contacts to clinics of hematology, oncology or nephrology, all within a 30-day window of the current admission.

Data on recent health care contacts were retrieved using the Danish National Patient Registry (DNPR) [13]. This register holds data on all citizens and permanent residents admitted to Danish hospitals since 1977 and all visits to hospital outpatient clinics since 1995. Each record includes the dates of hospital admission and discharge, up to 20 discharge diagnoses, and information on surgical procedures.

### Patients with chronic heart failure

Patients diagnosed with CHF at any time before the current admission were identified from the DNPR [13]. We defined CHF as a previous hospital discharge diagnosis or outpatient diagnosis of congestive heart failure, pulmonary edema with mention of heart failure, left ventricular failure, unspecified heart failure, cardiomyopathy, or hypertensive heart disease with congestive heart failure (with or without hypertensive renal disease or renal failure). CHF patients were further classified according to presence of CHF-related conditions: 1) cardiomyopathy (with or without any of the following diagnoses), 2) valvular heart disease (with or without any of the other diagnoses except cardiomyopathy), 3) previous myocardial infarction (with or without atrial fibrillation), 4) atrial fibrillation only, and 5) none of the above concomitant conditions. All diagnostic codes are provided in Additional file 2: Codes for diagnoses, procedures, medication, and blood tests.

Severity of CHF is not included in the diagnostic codes in the DNPR. Thus, as a proxy for CHF severity, patients were categorized according to daily dosage of redeemed prescriptions of loop-diuretics: non-users (no loop-diuretics), low dose (≤40 mg/day), medium dose (41–80 mg/day), high dose (81–159 mg/day), and very high dose (≥160 mg/day). We computed mean loop-diuretic dosages by dividing the number of dispensed tablets by a dispensing time interval of 180 days, as described previously [14, 15]. Loop-diuretic dosages have been shown to correlate positively with worsened New York Heart Association functional class and mortality risk, but not with glomerular filtration rate in CHF patients [15]. Data on filled prescriptions were retrieved from the Aarhus University Prescription Database (AUPD) [16], which holds data on redeemed prescriptions in the study area according to the Anatomical Therapeutic Chemical (ATC) classification system (ATC codes are provided in Additional file 2: Codes for diagnoses, procedures, medication, and blood tests). We calculated duration of CHF as the time elapsed between the first diagnosis of CHF and the sampling date of the first positive blood culture.

### Comorbidity, laboratory test results, and mortality

Data on sex, age, and marital status was retrieved from the Danish Civil Registration System, which is updated electronically on a daily basis and keeps track of demographic data and changes in vital status and migration for all Danish residents since 1968 [10, 11]. We computed a modified Charlson Comorbidity Index (m-CCI) using all available diagnoses registered in the DNPR up to 10 years before the current hospitalization excluding CHF from the index (the exposure variable of interest). The CCI is a validated comorbidity scoring system covering both the number and severity of 19 major disease categories [17, 18]. Patients were classified as having a low (score = 0), intermediate (score = 1–2), or a high comorbidity level (score >2). We further collated data on a number of conditions not included in the m-CCI, counting hypertension, drug- and alcohol-related conditions and dialysis (within 30 days of the current admission). Using the AUPD [16], we obtained data on the following filled prescriptions: Any previous use of antihypertensive treatment, statins (and other lipid lowering agents), anticoagulants, and use of immunosuppressant drugs, and antibiotics within 30 days of the SAB-related hospitalization (ATC codes are provided in Additional file 2: Codes for diagnoses, procedures, medication, and blood tests). The LABKA Database (CSC Scandihealth, Denmark) keeps laboratory test results from patients in Northern Denmark for the entire study period including the exact time of blood sample collection [19]. Using this database, we obtained information on white blood count levels on the date the first positive blood culture was drawn (laboratory codes are available in Additional file 2: Codes for diagnoses, procedures, medication, and blood tests). Data on all-cause mortality was retrieved from the Danish Civil Registration System [10, 11].

### Statistical analyses

All patients were followed from the date the first positive blood culture was drawn until death, emigration or 90 days, whichever came first. Patient characteristics (including demographics, comorbidity, and preadmission medication use) were tabulated according to CHF status. We computed the 90-day mortality risk using the Kaplan-Meier method (1 – survival function) and graphically displayed 90-day mortality for patients with and without CHF. Ninety-day mortality rates for patients with vs. without CHF were compared using a Cox proportional hazards model estimating hazard ratios as a measure of mortality rate ratios (MMRs) with corresponding 95 % confidence intervals (CIs). CHF exposure was further subcategorized according to CHF-related conditions, CHF severity and CHF duration. To examine whether mortality differed among subsets of CHF patients, we stratified the analyses by sex, age category (15–39, 40–59, 60–79, 80+ years), and m-CCI level (“low”, “intermediate”, and “high”). All MRRs were adjusted for age, sex, conditions included in the m-CCI, hypertension, alcohol related conditions, marital status (as a marker of socioeconomic status) and preadmission use of antibiotic therapy (within 30 days). The assumption of proportional hazards in the Cox models was assessed graphically and found appropriate. We conducted all statistical analyses using Stata 11.2 for Windows (Stata Corp, College Station, TX).

## Results

### Descriptive data

During the study period 2638 patients aged ≥15 years were hospitalized with incident CA-SAB, of which 390 (14.8 %) had CHF (Table 1). Median age was 77 (interquartile range (IQR), 70–82) and 67 (IQR, 54–78) years for patients with and without CHF, respectively. There were slightly more men among patients with CHF compared to patients without CHF (64.9 % vs. 60.6 %). Forty-eight percent of patients with CHF were classified as HCA vs. 41.4 % among patients without CHF. Methicillin-resistant *S.aureus* (MRSA) was rarely observed (0.5 % of all patients). Patients with CHF had considerably more hospital-diagnosed comorbidity than patients without CHF, including diabetes (31.0 % vs. 12.8 %), chronic pulmonary disease (30.8 % vs. 10.8 %), renal disease (33.3 % vs. 13.6 %), and hypertension (49.5 % vs. 20.4 %). Compared to patients without CHF, patients with CHF were more likely to have filled prescriptions for angiotensin-converting-enzyme inhibitors, beta blockers, acetylsalicylic acid, and statins.

**Table 1:** Characteristics of 2638 patients hospitalized with incident *Staphylococcus aureus* bacteremia in Northern Denmark, 2000-2011

	Patients with chronic heart failure	Patients without chronic heart failure
Numbers (%)	390 (14.8)	2248 (85.2)
Age, median (IQR)	76.6 (66.9–82.2)	67.4 (54.4–78.3)
15–39 years	12 (3.1)	221 (9.8)
40–59 years	48 (12.3)	557 (24.8)
60–79 years	194 (49.7)	988 (44.0)
≥ 80 years	136 (34.9)	482 (21.4)
Sex		
Men	253 (64.9)	1363 (60.6)
Women	137 (35.1)	885 (39.4)
*S.aureus* bacteremia		
Community-acquired	203 (52.1)	1320 (58.7)
Healthcare-associated	187 (48.0)	928 (41.3)
MRSA	3 (0.8)	10 (0.4)
Marital status		
Married	203 (52.1)	1067 (47.5)
Divorced or widowed	152 (39.0)	734 (32.7)
Never married	35 (9.0)	447 (19.9)
Selected comorbid conditions		
Diabetes mellitus	121 (31.0)	287 (12.8)
Peripheral vascular disease	99 (25.4)	229 (10.2)
Cerebrovascular disease	76 (19.5)	239 (10.6)
Chronic pulmonary disease	120 (30.8)	243 (10.8)
Moderate to severe renal disease	130 (33.3)	306 (13.6)
Hypertension	193 (49.5)	458 (20.4)
Conditions related to alcohol abuse	26 (6.7)	209 (9.3)
Conditions related to drug abuse	4 (1.0)	69 (3.1)
Dialysis within 30 days of admission	61 (15.6)	203 (9.0)
Modified Charlson Comorbidity Index		
Low (0)	42 (10.8)	720 (32.0)
Intermediate (1–2)	129 (33.1)	826 (36.7)
High (>2)	219 (56.2)	702 (31.2)
Preadmission medication use		
Immunosuppressive therapy^a^	3 (0.8)	25 (1.1)
Systemic antibiotic therapy^a^	82 (21.0)	454 (20.2)
ACE inhibitors^b^	298 (76.4)	788 (35.1)
Beta blockers^b^	271 (69.5)	764 (34.0)
Acetylsalicylic acid^b^	301 (71.2)	820 (36.5)
Statins^b^	174 (44.6)	451 (20.1)
Clinical biochemistry		
White blood count (10^9^/L)^c^		
< 3.5	11 (2.8)	100 (4.5)
3.5–10	52 (13.3)	440 (19.6)
> 10	262 (67.2)	1292 (57.5)
Unknown	65 (16.7)	416 (18.5)

*IQR* interquartile range, *MRSA* methicillin resistant *Staphylococcus aureus*, *ACE inhibitors* angiotensin-converting-enzyme inhibitors

^a^Any use within 30 days of the current admission. ^b^Any previous use prior to the current admission

^c^Measured on the date the first positive blood culture was drawn

### Ninety-day mortality

**Fig. 1:**
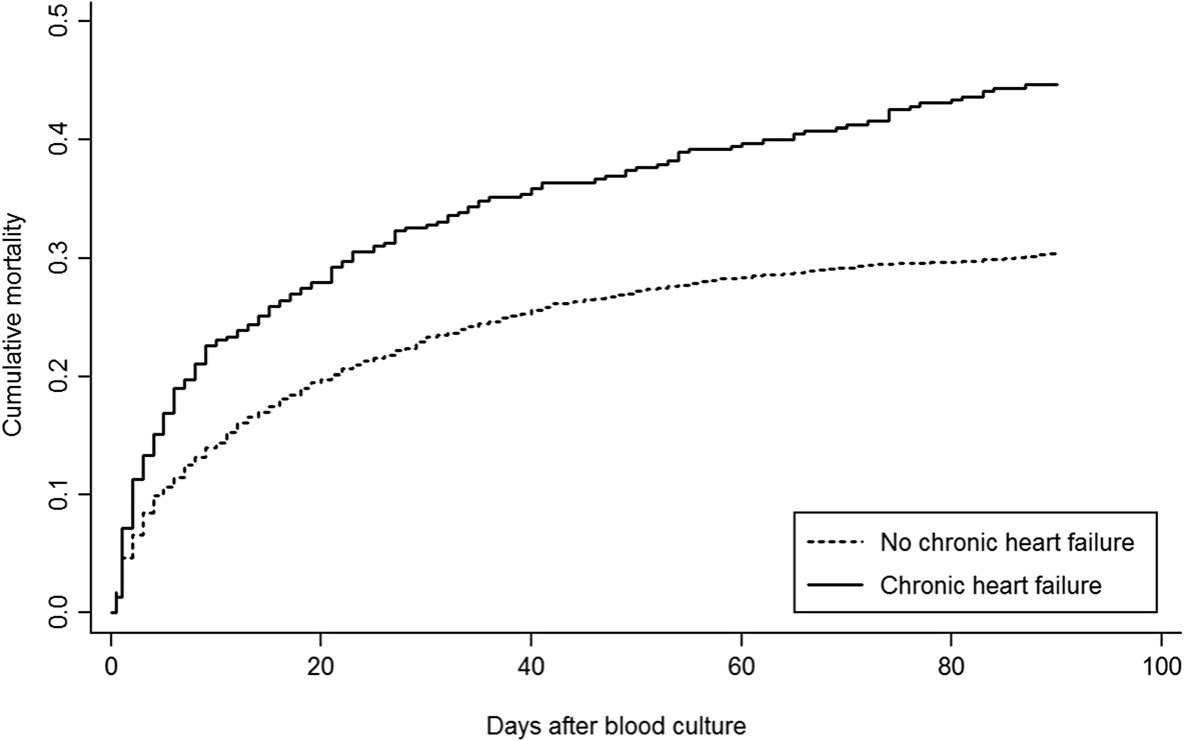
Cumulative mortality risk among incident *Staphylococcus aureus* bacteremia patients with and without chronic heart failure

Ninety-day cumulative mortality was 44.6 % in patients with CHF and 30.4 % in patients without CHF, respectively (Table 2 and Fig. 1). This yielded an unadjusted MRR of 1.60 (95 % CI, 1.36–1.89), and an adjusted MRR of 1.24 (1.04–1.48). Compared to 30.4 % among patients without CHF, 90-day mortality was 30.8 % among patients with concomitant cardiomyopathy (aMRR = 1.04 (95 % CI, 0.63–1.72)), 60 % among patients with a history of valve disease (aMRR = 1.73 (95 % CI, 1.26–2.38)), 41.2 % among patients with previous myocardial infarction (aMRR = 1.17 (95 % CI, 0.83–1.65)), and 41.0 % among CHF patients with none of the above concomitant conditions (aMRR = 1.12 (0.83–1.50)).

**Table 2:** Ninety-day mortality in incident *Staphylococcus aureus* bacteremia patients with versus without chronic heart failure (CHF)

	Number	Mortality % (95 % CI)	Crude MRR (95 % CI)	Adjusted^a^ MRR (95 % CI)
CHF				
Absent	2248	30.4 (28.6–32.4)	1.00 (ref.)	1.00 (ref.)
Present	390	44.6 (39.8–49.7)	1.60 (1.36–1.89)	1.24 (1.04–1.48)
CHF-related conditions				
CHF absent	2248	30.4 (28.6–32.4)	1.0 (ref.)	1.0 (ref.)
Cardiomyopathy	52	30.8 (20.1–45.2)	0.99 (0.60–1.62)	1.04 (0.63–1.72)
Valvular heart disease	70	60.0 (48.8–71.4)	2.44 (1.79–3.34)	1.73 (1.26–2.38)
Myocardial infarction	85	41.2 (31.6–52.4)	1.45 (1.04–2.05)	1.17 (0.83–1.65)
Atrial fibrillation	66	50.0 (38.7–62.5)	1.81 (1.27–2.57)	1.21 (0.85–1.73)
None of the above	117	41.0 (32.7–50.5)	1.46 (1.09–1.95)	1.12 (0.83–1.50)
CHF severity^b^				
CHF absent	2248	30.4 (28.6–32.4)	1.00 (ref.)	1.00 (ref.)
Non-users	99	32.2 (24.1–42.5)	1.08 (0.76–1.54)	0.99 (0.69–1.42)
Low dose (≤40 mg/day)	39	38.5 (25.3–55.5)	1.30 (0.78–2.16)	0.83 (0.50–1.40)
Medium dose (41–80 mg/day)	82	42.7 (32.8–54.1)	1.52 (1.08–2.14)	1.13 (0.80–1.59)
High dose (81–159 mg/day)	75	53.3 (42.6–64.9)	2.05 (1.49–2.83)	1.55 (1.11–2.14)
Very high dose (≥160 mg/day)	95	54.7 (45.1–64.9)	2.10 (1.59–2.79)	1.62 (1.21–2.18)
Duration of CHF				
CHF absent	2248	30.4 (28.6–32.4)	1.00 (ref.)	1.00 (ref.)
< 3 years	188	50.0 (43.1–57.3)	1.83 (1.48–2.27)	1.43 (1.14–1.78)
≥ 3- < 6 years	81	39.5 (29.8–51.0)	1.39 (0.98–1.99)	1.01 (0.71–1.46)
≥ 6- < 10 years	69	42.0 (31.4–54.5)	1.52 (1.05–2.21)	1.22 (0.84–1.78)
≥ 10 years	52	36.5 (25.1–51.1)	1.25 (0.79–1.97)	0.97 (0.61–1.54)

*CI* confidence interval, *MRR* mortality rate ratio

^a^Adjusted for age, sex, conditions included in the modified Charlson Comorbidity Index (excluding the condition in question), hypertension, alcohol related conditions, marital status, and antibiotic treatment within 30 days of admission

^b^Defined by daily loop-diuretic dosage

Compared to patients without CHF, an increased risk of death within 90 days was observed among patients with dosages of 81–159 mg/day (aMRR = 1.55 (95%CI, 1.11–2.14)) and ≥160 mg/day (1.62 (95 % CI, 1.21–2.18)), whereas no association was noted among patients with daily intakes of loop-diuretics ≤80 mg/day (Table 2). Ninety-day mortality was 50 % among patients with CHF of <3 years as compared to 30.4 % among patients with no CHF, corresponding to an aMRR of 1.43 (95 % CI, 1.14–1.78). Longer duration of CHF did not appear to be associated with a poor outcome: Thus, compared with patients with no history of CHF, the aMRR was 1.01 (95 % CI, 0.71–1.46) for ≥3- < 6 years of CHF, 1.22 (95 % CI, 0.84-1.78) for ≥6- < 10 years of CHF and 0.97 (95 % CI, 0.61–1.54) for ≥10 years of CHF history. We observed no consistent pattern or major differences in 90-day mortality according to sex, age, or m-CCI level (Table 3).

**Table 3:** Ninety-day mortality comparing incident community-acquired *Staphylococcus aureus* bacteremia in patients with and with chronic heart failure (CHF), stratified by sex, age, and modified Charlson Comorbidity Index level

	Patients without CHF	Patients with CHF	
	Mortality % (95 % CI)	Mortality % (95 % CI)	Adjusted^a^ MRR
Overall	30.4 (28.6–32.4)	44.6 (39.8–49.7)	1.2 (1.0–1.5)
Sex			
Male	27.8 (25.5–30.3)	38.7 (33.1–45.0)	1.2 (1.0–1.5)
Female	34.5 (31.4–37.7)	55.5 (47.4–63.9)	1.3 (1.0–1.7)
Age			
15–39 years	5.4 (3.1–9.4)	n/a^b^	n/a^b^
40–59 years	18.5 (15.5–22.0)	27.1 (16.7–42.0)	1.5 (0.8–2.7)
60–79 years	31.6 (28.8–34.6)	44.9 (38.2–52.1)	1.6 (1.2–2.0)
80+ years	53.3 (48.9–57.8)	54.4 (46.3–62.9)	1.1 (0.8–1.4)
Modified Charlson Comorbidity Index level			
Low (0)	23.9 (20.9–27.2)	42.9 (30.0–59.1)	1.2 (0.7–1.9)
Intermediate (1–2)	30.4 (27.4–33.7)	43.4 (35.4–52.4)	1.2 (0.9–1.6)
High (3+)	37.2 (33.7–40.9)	45.7 (39.3–52.5)	1.3 (1.0–1.7)

Reference group: patients without CHF

*CI* confidence interval, *MRR* mortality rate ratio

^a^Adjusted for age, sex, conditions included in the modified Charlson Comorbidity Index (excluding CHF), hypertension, alcohol related conditions, marital status, and antibiotic treatment within 30 days of admission

^b^Not applicable due to absence of events

## Discussion

In this large cohort study of 2638 patients with incident SAB, we observed a 24 % increase in 90-day all-cause mortality associated with CHF. Compared to patients without CHF, the excess risk of death within 90 days was most pronounced among CHF patients with concomitant valvular disease, patients with CHF of less than 3 years duration, and patients with a daily loop-diuretic dosage above 80 mg/day.

Our results are in line with the limited existing knowledge on the impact of CHF on mortality in SAB patients [6–9]. In a Norwegian cohort study of 374 patients with SAB, Paulsen et al. [6] observed an age- and sex-adjusted odds ratio (OR) of 2.4 (95 % CI, 1.21–4.80) for 30-day mortality comparing patients with and without CHF. In a Swiss cohort study [9] including 308 SAB patients from a single referral center, the authors observed an unadjusted OR of 2.4 (95 % CI, 1.0–5.6) of death within 90 days associated with CHF. A Columbian cohort study [7] examining risk factors of 90-day mortality in 267 cancer patients with SAB reported a hazard ratio of 10.6 (95 % CI, 1.8–63.7) comparing patients with and without CHF. Finally, a Taiwanese cohort study of 227 patients with persistent MRSA-SAB [8], found an 30-day mortality OR of 2.85 (95 % CI, 1.44–5.65) for patients with CHF compared to patients without. However, several issues should be taken into account when interpreting the results of these previous studies: Small and selected study populations [7, 8], limited numbers of patients with CHF (n < 70) [6–9], and insufficient adjustment for concomitant comorbid conditions [8, 9], could partly explain the findings. Moreover, in contrast to our study, none of the previous studies investigated the impact of CHF on mortality according to CHF-related conditions, CHF severity or duration of CHF [6–9].

Several mechanisms may underlie our observations. Myocardial dysfunction is a well-known complication of sepsis [20, 21]. Cardiac dysfunction in patients with sepsis is characterized by ventricular dilatation, decreased ejection fraction, and blunted ability to increase cardiac output despite elevated catecholamine levels [20]. Patients with CHF may be especially susceptible to these mechanisms, which could partly explain our observed difference in mortality among patients with versus without this underlying condition. Still, patients with CHF were older, more frequently men, and had more comorbidity registered than those without CHF, all of which are important prognostic factors in patients with SAB [2]. Adjusting for these factors in our model attenuated the association between CHF and mortality suggesting that a considerable part of the high mortality associated with SAB is conveyed by the combined burden of age, sex and comorbidity. In our study, the increased 90-day mortality associated with CHF was most pronounced among CHF patients with valvular heart disease and among patients with short duration of CHF. CHF patients with concomitant valvular heart disease may in particular be at high risk of pulmonary edema and circulatory collapse secondary to sepsis. However, valvular heart disease represents a major risk factor for infective endocarditis [22], which may add to the poor prognosis of these patients. The mechanisms underlying the increased risk of death among patients with short duration of CHF remain unclear and may most likely be multifactorial. It is possible, however, that patients with shorter duration of CHF differ from patients with long CHF duration with regards to CHF management and clinical stability which may influence the outcome from CA-SAB.

Our study has several strengths including its size, population-based design and adjustment for relevant confounders facilitated by our access to medical databases ensuring a complete prescription and hospitalization history. All data was collected prospectively and independently of the study hypothesis, thus reducing the risk of selection and information biases, and follow-up was virtually complete. However, some important limitations should be addressed in the interpretation of our results. Identification of patients with CHF from medical databases may be hampered by inaccurate coding, which would bias our results towards unity. Yet, two recent Danish validation studies reported positive predictive values for chronic heart failure in the DNPR of 81 % [23] and 100 % [19], respectively. Physicians may be more likely to admit patients with CHF on suspicion of infection compared to patients without CHF. Such surveillance bias would induce an underestimation of the relative risk associated with SAB. However, white blood counts of patients with and without CHF were comparable, and the proportions of patients who had received antibiotics prior to the current admission were almost similar. In addition, we observed no substantial differences in the proportions of patients classified as HCA-SAB among the two groups. This argues against, but does not preclude notable bias associated with the triage and treatment of patients with CHF in our study. On the other hand, the clinical management of patients with CA-SAB was not standardized across hospitals, which might have influenced our results. Furthermore, we lacked data on infective foci including venous catheters and other vascular access devices, which have been associated with SAB prognosis in several prior studies [1, 2].

We used loop-diuretic dosage as a proxy for CHF severity, since we did not have access to data on ejection fraction or New York Heart Association Functional Class among patients with CHF. If some patients used loop-diuretics for other reasons than CHF (e.g., concomitant renal failure) this may have led us to underestimate any differences between less severe and severe CHF, although we do not expect this to alter our overall conclusions. Finally, the medical databases did not contain data on smoking and obesity, still these potential confounders may be partly accounted for by adjustment for lifestyle-associated comorbidities included in our statistical models.

Due to the low prevalence of MRSA in our study area [24], these data and results may not be directly applicable to settings with higher MRSA prevalence. Still, our results may most likely be applicable to other healthcare systems with equal unfettered access to medical care and prescription medication including CHF drugs.

## Conclusion

In summary, patients with CA-SAB and CHF experienced higher 90-day mortality than patients without CHF, which was most apparent among CHF patients with valvular heart disease, patients with a short history of CHF, and patients with high daily dosages of loop-diuretics. SAB patients with CHF may benefit from the collaborated care of infectious diseases specialists and cardiologists ensuring increased adherence to evidence-based guidelines, optimized post-discharge follow-up and possibly improved clinical outcomes.

## Abbreviations

ATC codes, anatomical therapeutic chemical classification system codes; AUPD, Aarhus University prescription database; CA-SAB, community-acquired *Staphylococcus aureus* bacteremia; CHF, chronic heart failure; CI, confidence interval; DNPR, danish national patient registry; HCA-SAB, healthcare-associated *Staphylococcus aureus* bacteremia; IQR, interquartile range; m-CCI, modified Charlson comorbidity index; MRR, mortality rate ratio; MRSA, methicillin-resistant *Staphylococcus aureus*; OR, odds ratio; SAB, *Staphylococcus aureus* bacteremia.

## Additional files

Additional file 1: Identification and susceptibility testing of *S.aureus* isolates (PDF 122 kb) Additional file 2: Codes for diagnoses, procedures, medication and blood tests (PDF 27 kb)
