# Supplementary material for: Chronic heart failure and mortality in patients with community-acquired Staphylococcus aureus bacteremia: a population-based cohort study
Source: BMC Infect Dis. 2016 May 25;16:227. doi: 10.1186/s12879-016-1570-7 (PMC4880885; doi:10.1186/s12879-016-1570-7)
Supplement: Additional file 2: — Codes for diagnoses, procedures, medication and blood tests (PDF 27 kb) [file 12879_2016_1570_MOESM2_ESM.pdf]

Additional file 2

Codes for diagnoses, procedures, medication and blood tests

Preadmission comorbid conditions. Diagnoses codes are according to the 8th and 10th revision of the International Classification of Diseases, ICD-8 and ICD-10

**Condition**	**ICD-8**	**ICD-10**
Chronic heart failure	427.09, 427.10, 427.11, 427.19, 428.99, 784.29	I50, I11.0, I13.0, I13.2, I42.0, I42.6, I42,7, I42.8, I42.9, I25.5
Cardiomyopathy	425.99	I42
Heart valve disease	394.00-394.02, 394.08, 394.90, 394.91, 394.92, 394.98, 394.99, 395.90, 395.01, 395.02, 395.08, 395.90-395.92, 395.98-395.99, 396.00-396.04, 396.08-39.609, 396.90-396.93, 396.04, 396.08, 396.09, 396.90, 396.91-396.94, 396.98-397.01, 397.09, 398.99	I34, I35, I05-I08, I09.1, I09.8, I09.9, I36-I39
Myocardial infarction	410.09, 410.99	I21-I23
Atrial fibrillation	427.93, 427.94	I48
Diabetes	249.00, 249.06, 249.07, 249.09, 250.00, 250.06, 250.07, 250.09	E10-E14. O24 (except O24.4), G63.2, H36.0, N08.3
Peripheral vascular disease	440, 441, 442, 443, 444, 445	I70, I71, I72, I73, I74, I77
Cerebrovascular disease	430-438	I60-I69, G45, G46
Dementia	290.09-290.19, 293.09	F00-F03, F05.1, G30

Chronic pulmonary disease	490-493, 515-518	J40-J47, J60-J67, J68.4, J70.1, J70.3, J84.1, J92.0, J96.1, J98.2, J98.3
Connective tissue disease	712, 716, 734, 446, 135.99	M05, M06, M08, M09, M30, M31, M32, M33, M34, M35, M36, D86
Ulcer disease	530.91, 530.98, 531-534	K22.1, K25-K28
Mild liver disease	571, 573.01, 573.04	B18, K70.0-K70.3, K70.9, K71, K73, K74, K76.0
Hemiplegia	344	G81, G82
Moderate to severe renal disease	403, 404, 580-583, 584, 590.09, 593.19, 753.10-753.19, 792	I12, I13, N00-N05, N07, N11, N14, N17-N19, Q61
Any tumor	140-194	C00-C75
Leukemia	204-207	C91-C95
Lymphoma	200-203, 275.59	C81-C85, C88, C90, C96
Moderate to severe liver disease	070.00, 070.02, 070.04, 070.06, 070.08, 573.00, 456.00-456.09	B15.0, B16.0, B16.2, B19.0, K70.4, K72, K76.6, I85
Metastatic solid tumor	195-198, 199	C76-C80
AIDS	079.83	B21-B24
Hypertension	400-404	I10-I13
Osteoporosis	723.09	M80-M82

Conditions related to alcohol abuse	291.09-291.99, 303.09-303.29, 303.91-303.99	F10, K86.0, Z72.1. T51, K29.2, G62.1, G31.2, I42.6, K70
Conditions related to drug abuse	304.09-304.99	F11-F16, F18-F19, T40

Procedures. Codes regarding dialysis are according to Danish Treatment codes and according to the 10th revision of the International Classification of Diseases, ICD-10.

	Danish treatment codes	ICD-10
**Dialysis**	98300, 94340, 94350<br>BJFD0, BJFD2	Z99.2, Z49, Z49.2, BJFD
**Surgery**	All surgical codes (K-codes) in the Nordic Medico-Statistical Committee (NOMESCO) Classification of Surgical Procedures.	

Medication codes are according to the Anatomical Therapeutic Classification (ATC)

Type of medication	ATC codes
Loop-diuretics	C03CA01
Immunosuppressive therapy	L01, L04
Systemic antibiotic therapy	J01
ACE inhibitors	C09
Beta-blockers	C07
Acetylsalicylic acid	B01AC06
Statins	C10AA, C10B, B04AB,

Blood tests according to local analysis codes and Nomenclature for Properties and Units (NPU)-codes

Blood test	Local analysis and NPU-codes
White blood count	NPU02593, 2593, 122577, 37, 1312240, 141240
